# Patriotism and Nurse-Training Schools

**Published:** 1916-10-21

**Authors:** 


### PATRIOTISM AND NURSE-TRAINING SCHOOLS.

### IX. Royal Infirmary, Edinburgh.

The R.I.E. nurses engaged in war nursing are in truth a goodly company. The size of this institution, second to none in the kingdom, and the number of nurses in training and permanently engaged within its walls have enabled it to perform exceptional services for the common cause.

In the first section are nurses sent out by Miss Gill as members of the R.I.E. Civil Reserve, the majority direct from hospital. Of these Miss S. McIntosh has been awarded the R.R.C., First Class, and Miss J. M. Osler and Miss M. Taylor, A.R.R.C., Second Class. Miss C. B. Robb has had the honour of being twice mentioned in dispatches for her valuable services, and Miss E. Tully and Miss A. Weatherstone have also been mentioned. The other members of the R.I.E. Civil Reserve serving in military hospitals abroad are as follows:—Nurses H. Addison, H. B. Anderson, Margaret Anderson, M. M. Anderson, M. V. Bean, A. I. Burton, A. Cairns, B. M. Cameron, E. Cameron, J. W. Cameron, M. A. Campbell, J. A. Cassels, C. S. Cathels, I. M. Chalmers, A. Cockburn, M. Cook, H. Crawford, A. Cumming, K. M. Dill, A. Douglas, L. Dow-Sainter, M. E. Edwards, W. A. Evers, M. R. Fairbairn, A. C. Felpts, A. C. Ferrier, A. A. Forbes, M. J. Gerard, I. Gibson, M. J. Gow, L. E. Grant, C. Gray, M. Hay, J. Ingram, M. M. Kerr, J. T. Leechman, K. E. Leith, A. M. Locke, H. Mackay, C. Macleod, I. Melville, D. Miller, E. Millican, E. Milligan, S. P. Morris, S. Munro, J. A. Nairn, E. A. Nicholson, K. O'Donoghue, E. Park, M. B. Peterkin, G. Phillips, M. Robertshaw, E. Scofield, M. M. Sinclair, D. S. Smith, M. E. Thomson, M. M. Urquhart, A. E. Walton, M. Webster, I. M. Welsh, H. W. Westwater, E. A. Williams, H. Wilson, I. Wilson, M. T. Wilson, S. McKinnon, L. Henderson, M. A. Macdonald, H. Higgins, M. Anderson, J. A. McAndrew, E. J. Stopani, M. Storrie, J. B. Inglis, J. B. May, M. A. M. Robertson, J. S. Brotchie, E. M. Thomson, M. Murray, A. Ferguson, B. D. Reid, E. J. Gordon, and A. T. Nicol.

Next in order must be mentioned the nurses who left to join various other branches of the Service direct from the Royal Infirmary:—

*Queen Alexandra Imperial Military Nursing Service Reserve*: Sister J. Livingston, Nurses H. Paterson, I. Scott, H. Thom, Skene, L. McKinnon, M. A. Munro, and M. F. Smith.

*Territorial Force Nursing Service*: Miss C. W. Millar (assistant superintendent), Sister Laing (twice mentioned in dispatches) and Sister Murray (once mentioned), Sisters Richard, Sloan, Totton, Gardner, G. Smith, and Lamb. Nurses M. W. Dewar, E. D. Henderson, A. Lorimer, I. S. Macdougall, M. Maclean, M. McQueen, H. Mair, E. Millar, H. I. Nicholson, H. W. Ramsay, J. Smith, M. Evans, A. Aitken, E. D. Swinton, M. Morrison, and D. Allan.

*War Hospitals*: Miss E. J. Cumming (assistant superintendent), Miss I. E. Bodin (who has been made Associate of the Royal Red Cross), Nurses J. Rae, Hansell, Wittet, C. D. Keltie, A. Reid, Hutchison, Farquharson, R. Paterson, G. Ross, and A. Fraser.

The list of nurses now serving in the war who hold the certificate of the Royal Infirmary, Edinburgh, is too remarkable to be passed over, though space prevents us from mentioning more than a few of the names it contains. Thirteen members of Q.A.I.M.N.S. are among the number, of whom the following have been honoured with the Order of the Royal Red Cross:—Misses H. W. Reid, J. Hoadley, Nixon, J. E. Dods, A. F. Byers, A. B. Cameron, and G. H. Sellar. Forty-one R.I.E. nurses are in Q.A.I.M.N.S.R., and of these Misses K. L. Bulman, A. J. Williams, and A. L. Wilson have been honoured with the Order of the Royal Red Cross, while Miss M. J. Kirkpatrick has been made A.R.R.C.

In the Territorial Nursing Service the list is headed by the Principal Matron, Miss A. W. Gill, R.R.C., and includes seventy other past members of the famous training-school. A good number of these have distinguished themselves and earned recognition. Misses L. Edmonson, M. Sinclair, and E. D. Smaill have been made members of the Royal Red Cross; Misses A. P. Douglas, A. Leslie, and C. Yule have been made Associates of the Order; Misses C. Carnegie and M. Pool have been mentioned in dispatches.

The R.I.E. nurses serving in auxiliary and other war hospitals at home and abroad number forty-six. Six are serving in the French Flag Nursing Corps, one is in the Canadian Army Nursing Corps. Miss B. B. Bowhill and Miss E. K. Philp served as matrons in the Scottish Women's Hospital in Serbia. Two of the sisters in charge of military wards in the Royal Infirmary have also been decorated with the Royal Red Cross, Second Class; Miss F. McKinnon and Miss S. Elliott.

The Roll of Honour comprises Miss J. B. Smith, R.R.C., who died while acting as matron at a military hospital in France; Miss Harrower, assistant matron at Epsom War Hospital; and Miss M. T. Kerr, staff nurse with the 2nd Scottish General Hospital.

The lists testify to the admirable *esprit de corps* among the R.I.E. nurses, which enables the matron to follow their career and register their movements. We may add that as they first reached us the names of the hospitals where each member was serving was appended. The frequent changes entailed by the war have, however, rendered it safer in the interests of accuracy not to mention localities.

For Matrons' and Sisters' Appointments, see p. 56. For Mental Nurses in War Hospitals, see p. 55.

